# Synthesis, crystal structure and anti­cancer activity of the complex chlorido­(η^2^-ethyl­ene)(quinolin-8-olato-κ^2^ *N*,*O*)platinum(II) by experimental and theoretical methods

**DOI:** 10.1107/S2056989024003748

**Published:** 2024-04-30

**Authors:** Nguyen Thi Thanh Chi, Ngo Tuan Cuong, Tran Thu Trang, Pham Van Thong, Nguyen Thi Bang Linh, Nguyen Thi Khanh Ly, Luc Van Meervelt

**Affiliations:** a Department of Chemistry, Hanoi National University of Education, 136 Xuan Thuy, Cau Giay, Hanoi, Vietnam; b R&D Center, Vietnam Education and Technology Transfer JSC, Hanoi, Vietnam; c Department of Chemistry, KU Leuven, Biomolecular Architecture, Celestijnenlaan 200F, Leuven (Heverlee), B-3001, Belgium; Harvard University, USA

**Keywords:** platinum(II) complex, crystal structure, anti­cancer activity, 8-hy­droxy­quinoline, DFT

## Abstract

The complex [Pt(C_9_H_6_NO)Cl(C_2_H_4_)], (I), was synthesized and structurally characterized by ESI mass spectrometry, IR, NMR spectroscopy, DFT calculations and X-ray diffraction. The results showed that the deprotonated 8-hy­droxy­quinoline (C_9_H_6_NO) coordinates with the Pt^II^ atom *via* the N and O atoms while the ethyl­ene coordinates in the η^2^ manner and in the *trans* position compared to the coordinating N atom. The crystal packing is characterized by C—H⋯O, C—H⋯π, Cl⋯π and Pt⋯π inter­actions. Complex (I) showed high selective activity against Lu-1 and Hep-G2 cell lines with IC_50_ values of 0.8 and 0.4 µ*M*, respectively, 54 and 33-fold more active than cisplatin. In particular, complex (I) is about 10 times less toxic to normal cells (HEK-293) than cancer cells Lu-1 and Hep-G2. Furthermore, the reaction of complex (I) with guanine at the N7 position was proposed and investigated using the DFT method. The results indicated that replacement of the ethyl­ene ligand with guanine is thermodynamically more favorable than the Cl ligand and that the reaction occurs *via* two consecutive steps, namely the replacement of ethyl­ene with H_2_O and the water with the guanine mol­ecule.

## Abstract 2

In the complex [Pt(C_9_H_6_NO)Cl(C_2_H_4_)], ethyl­ene coordinates with Pt^II^ in the η^2^ manner and in the *trans* position compared to the coordinating N atom. The *in vitro* anti­cancer activity was investigated and DFT calculations were performed to support its anti­cancer activity.

## 1. Chemical context

8-Hy­droxy­quinoline (C_9_H_6_OH) and its complexes are well-known heterocyclic compounds in the pharmaceutical field due to their excellent biological activities (Song *et al.*, 2015 ▸; Cherdtrakulkiat *et al.*, 2016 ▸; Oliveri & Vecchio, 2016 ▸; Gupta *et al.*, 2021 ▸; Prachayasittikul *et al.*, 2013 ▸; Bissani Gasparin & Pilger, 2023 ▸). Recently, many complexes of the type [Pt(C_9_H_6_O)Cl(*L*)] (*L* = aryl­olefin, dimethyl sulfoxide, 1,3,5-tri­aza-7-phosphaadamantane) have been synthesized and tested for *in vitro* activity on many human cancer cell lines (Da *et al.*, 2015 ▸; Thanh Chi *et al.*, 2017 ▸; Nguyen Thi Thanh *et al.*, 2017 ▸; Chi *et al.*, 2018 ▸; Živković *et al.*, 2018 ▸; Yang *et al.*, 2023 ▸; Meng *et al.*, 2016 ▸). The results illustrated that most of the complexes showed high activity on the tested cell lines. However, the crystal structure and anti­cancer activity of the simplest olefin-containing complexes and 8-hy­droxy­quinoline derivative have less information available (Al-Najjar & Al-Lohedan, 1990 ▸).

**Figure 1:**
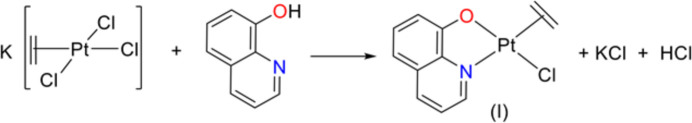
Synthesis of complex [Pt(C_9_H_6_O)Cl(C_2_H_4_)] (I) from Zeize’s salt and 8-hy­droxy­quinoline.

Complex [Pt(C_9_H_6_O)Cl(C_2_H_4_)] (I) was synthesized by the reaction between Zeise’s salt and 8-hy­droxy­quinoline in ethanol/water solvent with the molar ratio of Zeise’s salt:8-hy­droxy­quinoline being 1:1 (Fig. 1 ▸). The reaction was carried out at ambient temperature and complex (I) was formed in a high yield of 90% within around 3 h. 
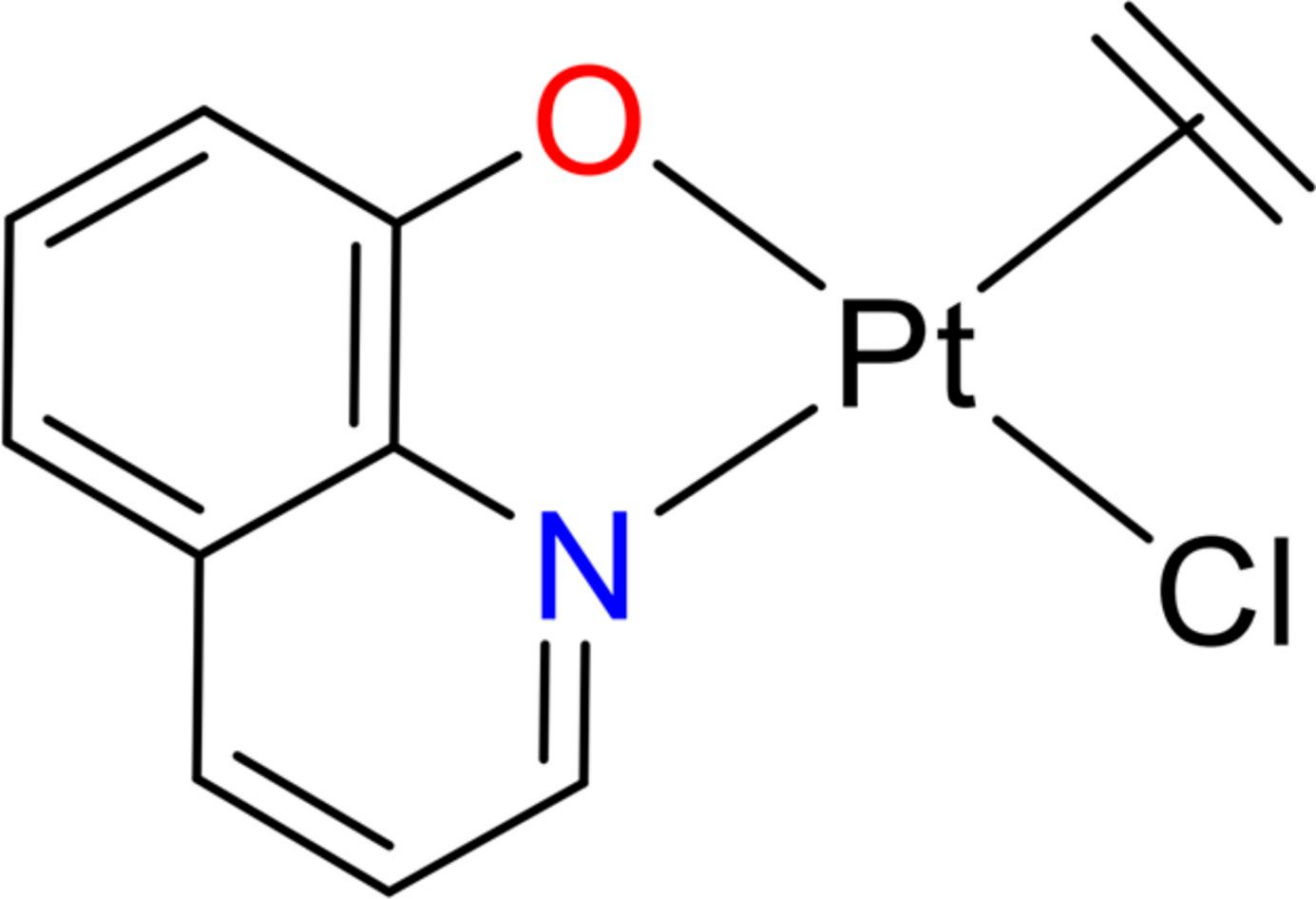


In the negative-mode ESI-MS spectrum of (I), a base peak with the correct isotopic pattern for [PtCl_3_]^−^ was observed (Fig. S1). This anion was formed as complex (I) released the C_2_H_4_ and C_9_H_6_NO ligands and added two Cl^−^ ions. Based on the IR spectrum (Fig. S2), it is not unequivocally possible to confirm the deprotonation of the OH group of 8-hy­droxy­quinoline since the absorption band characteristic of ν_OH_ around 3500 cm^−1^ decreased only slightly compared to the free ligand. In the ^1^H NMR spectrum of (I), the resonance signal at 4.90 ppm with an intensity of 4H corresponds to the ethyl­enic protons (Fig. S3). Upon coordination to Pt^II^, this signal has clear ^195^Pt satellites with ^2^ *J*_PtH_ = 60 Hz and shifts upfield in comparison to that of non-coordinated ethyl­ene (5.28 ppm; König *et al.*, 2012 ▸). Moreover, the presence of ^195^Pt satellites at the signal of the proton, which is two sigma bond distances away from the N atom, at 9.11 ppm with ^3^ *J*_PtH_ = 35 Hz and the absence of signal for the OH group in the spectrum are evidence for the coordination of deprotonated 8-hy­droxy­quinoline with Pt^II^ through both the N and O atoms. Notably, the chemical shift δ of the ethyl­ene protons in complex (I) shifts downfield compared to that in the Zeise’s salt (4.246 ppm; König *et al.*, 2012 ▸), demonstrating that the C_9_H_6_NO ligand has weakened the Pt—(C=C) bond in complex (I). In other words, the bond order of ethyl­ene decreases in the following order: free ethyl­ene > complex (I) > Zeise’s salt. This conclusion is further strengthened by comparing the C=C bond distances in free ethyl­ene, complex (I) and Zeise’s salt (Black *et al.*, 1969 ▸), which are 1.34, 1.379 (10) and 1.44 Å, respectively. In the NOESY spectra (Fig. S4), there is no appearance of a cross peak between the protons of ethyl­ene and the protons of 8-hy­droxy­quinoline. This suggests that the nitro­gen heteroatom of 8-hy­droxy­quinoline and the ethyl­ene are not *cis* but *trans* to one another in the Pt^II^ coordination sphere.

## 2. Structural commentary

**Figure 2:**
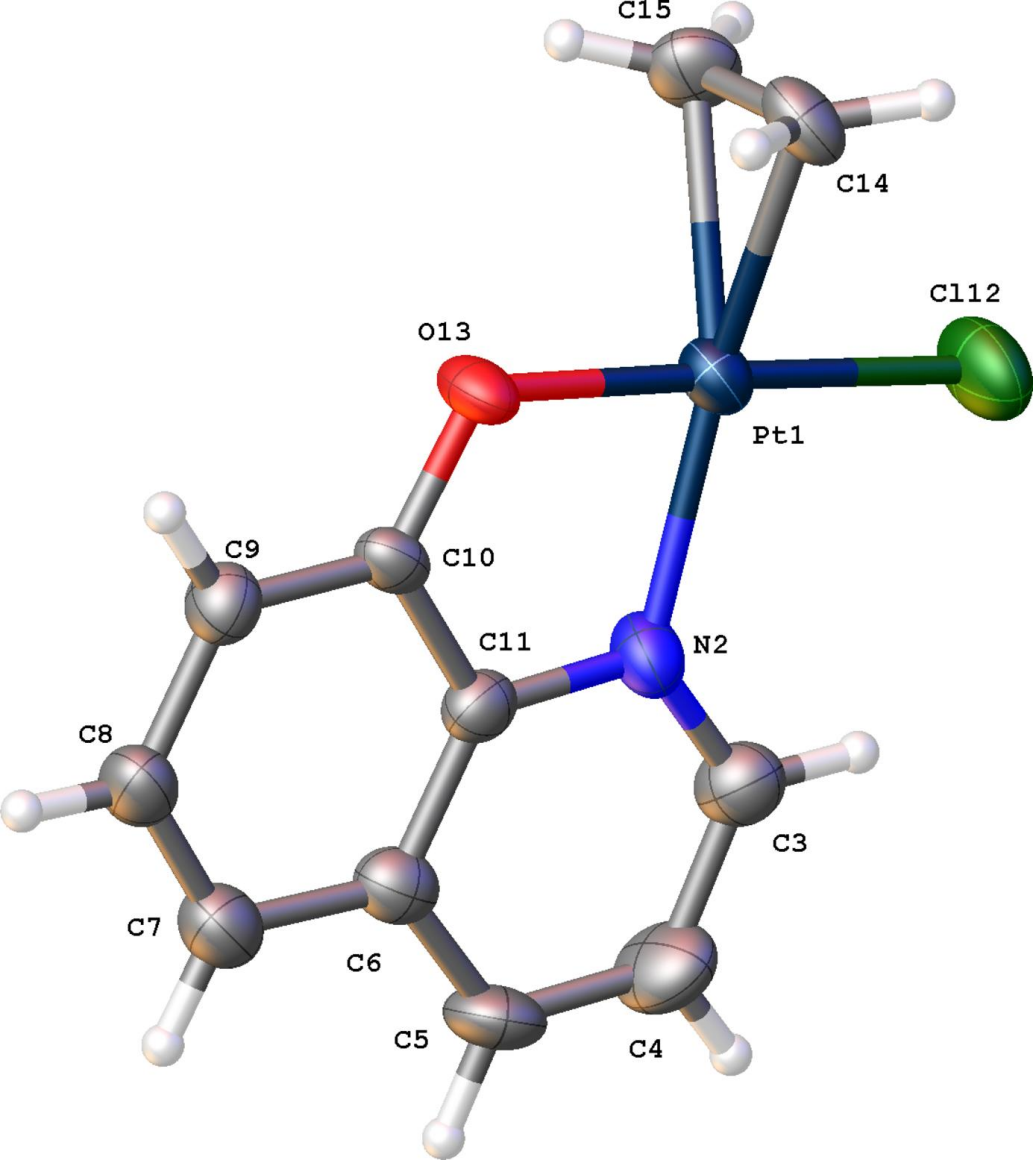
The mol­ecular structure of complex (I), showing the atom-labelling scheme. Displacement ellipsoids are drawn at the 50% probability level.

Complex (I) crystallizes in the monoclinic space group *P*2_1_/*c* with one mol­ecule in the asymmetric unit (Fig. 2 ▸). The central Pt^II^ atom displays a distorted square-planar coordination with one Cl atom, the N and O atoms of quinolin-8-olate and the C=C double bond as the coordination sphere. The Pt^II^ atom deviates by 0.020 (3) Å from the best plane through atoms N2, Cl12, O13 and the mid-point of the double bond (r.m.s. deviation = 0.012 Å). The C=C double bond and N atom are *trans* with respect to each other. The deviations of atoms Pt1, Cl12 and O13 with respect to the planar quinoline ring (r.m.s. deviation = 0.013 Å) are −0.131 (1), −0.263 (2) and −0.026 (4) Å, respectively. The virtual three-membered ring Pt1–C14–C15 makes an dihedral angle of 86.9 (5)° with the quinoline plane. A short intra­molecular C3—H3⋯Cl12 contact is observed (H3⋯Cl12 distance = 2.82 Å).

## 3. Supra­molecular features

**Figure 3:**
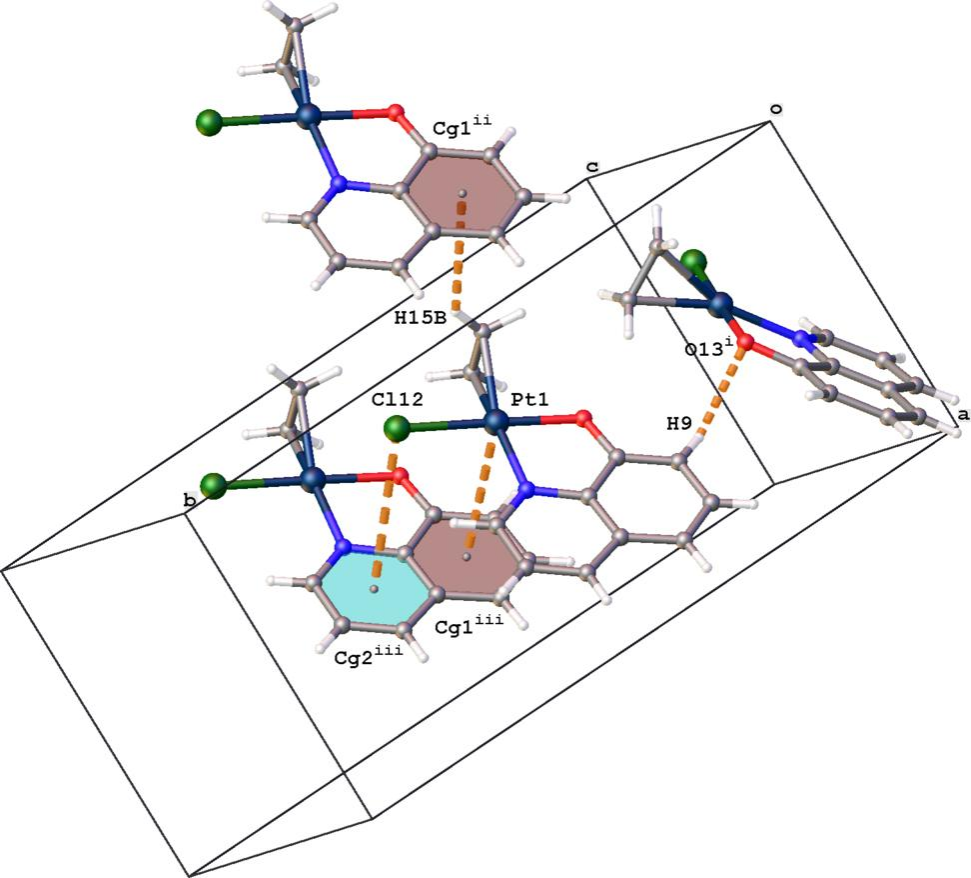
Partial packing diagram for (I) showing the C—H⋯O, C—H⋯π, Cl⋯π and Pt⋯π inter­actions (orange dashed lines). *Cg*1 and *Cg*2 are the centroids of rings C6–C11 (brown) and N2/C3–C6/C11 (blue), respectively. [Symmetry codes: (i) *x*, −*y* + 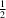 , *z* − 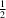 ; (ii) *x* − 1, *y*, *z*; (iii) *x*, *y*, *z* + 1.]

The crystal packing is mainly built up by C—H⋯O and C—H⋯π inter­actions (Table 1 ▸, Fig. 3 ▸). One of the quinoline H atoms (H9) forms a C—H⋯O hydrogen bond with the quinolin-8-olate O atom of an adjacent complex related by a *c*-glide plane [H9⋯O13^i^ = 2.58 Å; symmetry code: (i) *x*, −*y* + 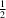 , *z* − 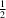 ]. One of the ethyl­ene H atoms (H15*B*) inter­acts with the C6–C11 aromatic ring, which results in chain formation in the *a*-axis direction [H15*B*⋯*Cg*1^ii^ = 2.95 (6) Å; *Cg*1 is the centroid of the C6–C11 ring; symmetry code: (ii) *x* − 1, *y*, *z*]. Furthermore, the packing shows chain formation in the *c*-axis direction as a result of Cl⋯π and Pt⋯π inter­actions [Cl12⋯*Cg*2^iii^ = 3.948 (4) Å; Pt1⋯*Cg*1^iii^ = 3.647 (3) Å; *Cg*2 is the centroid of the N2/C3–C6/C11 pyridine ring; symmetry code: (iii) *x*, *y*, *z* + 1].

## 4. Database survey

A search of the Cambridge Structural Database (CSD, Version 5.45, update of March 2024; Groom *et al.*, 2016 ▸) for Pt complexes coordinated to Cl, N, O and C=C resulted in ten hits. The average Pt—Cl (2.289 Å), Pt—N (2.060 Å) and Pt—O (2.012 Å) distances agree well with the distances in (I), which are 2.2951 (18) Å, 2.041 (5) Å and 2.004 (4) Å, respectively. The average distance between Pt and the mid-point of the C=C double bond of 2.040 Å is also comparable with the equivalent distance of 2.023 (5) in (I).

Except for chloro-(penta­fluoro­phenolato)(η^2^-*o*-vinyl-*N*,*N*-di­methyl­aniline)platinum(II) (refcode PFPVAP; Cooper *et al.*, 1978 ▸) and *cis*-chloro­(sarcosine-*N*,*O*)-(η^2^-2-methyl-3-buten-2-ol)platinum(II) (SOLCAX; Erickson *et al.*, 1991 ▸), the double bond and the N atom are in a *trans* position with respect to each other.

Similar to the title compound, the N and O atoms are part of 8-hy­droxy­quinoline in three structures: chloro­(5,7-di­chloro­quinolin-8-olato){2-meth­oxy-4-[prop-2-en-1-yl]phenol}platin­um(II) (SEMXEQ; Nguyen Thi Thanh *et al.*, 2017 ▸), chloro­(prop­yl{2-meth­oxy-4-[prop-2-en-1-yl]phen­oxy}acetate)(quinolin-8-olato)platinum(II) (HISBAP; Chi *et al.*, 2018 ▸) and chloro­(propan-2-yl{2-meth­oxy-4-[prop-2-en-1-yl]phen­oxy}acetate)(quinolin-8-olato)platinum(II) (HISBET; Chi *et al.*, 2018 ▸).

For 1095 Pt complexes with a double bond as a ligand for Pt, the average distance from Pt to the mid-point of the double bond is 2.071 Å, with minimum and maximum values 1.837 and 2.435 Å, respectively.

## 5. *In vitro* cytotoxicity

The *in vitro* anti­cancer activity of complex (I) was investigated on four human cancer cell lines, namely KB, Hep-G2, Lu-1, and MCF-7 and the normal cell line HEK-293. The results in Table 2 ▸ show that complex (I) exhibits significant activity against the Lu-1 and Hep-G2 cell lines with IC_50_ values of 0.8 and 0.4 µ*M*, respectively, 54 and 33-fold more active than cisplatin. Compared to the series of complexes [Pt(C_9_H_6_NO)Cl(aryl­olefin)] (aryl­olefin = safrole, eugenol, methyl­eugenol, prop­yl/isopropyl eugenoxyacetate), complex (I) shows equivalent activity but is more selective on the Lu-1 and Hep-G2 cell lines. Remarkably, complex (I) is approximately 10 times less toxic to normal cell (HEK-293) than cancer cells Lu-1 and Hep-G2.

## 6. Density function theory calculations

To provide information supporting the experimental study of the anti­cancer activity of complex (I), we performed several quantum chemical calculations using density functional theory (DFT), which is implemented in the *Gaussian 09* program package (Frisch *et al.*, 2016 ▸). Firstly, the geometric structure of complex (I) was optimized, followed by the frequency calculation, to ensure that the obtained structure was a minimum energy structure. The long-range corrected version of Becke Three-Parameter Hybrid Functionals (B3LYP) by Handy and colleagues using the Coulomb attenuation method, CAM-B3LYP (Yanai *et al.*, 2004 ▸) was used. The contracted Gaussian basis sets with polarization and diffuse functions 6-311+G(d) (McLean & Chandler, 1980 ▸) were used for C, H, O, N, Cl atoms and the Dunning’s correlation consistent basis sets, also with diffuse functions Aug-cc-pVDZ-PP was used for the Pt atom (Pritchard *et al.*, 2019 ▸). The optimized structure is shown in Fig. S5. The bond lengths and bond angles of the coordination environment calculated by the DFT and determined by the XRD of complex (I) show a good agreement (Table S1). This also indicates that the CAM B3LYP//6-31+G(d)/ccpVDZ-PP method is suitable for investigating the complex.

Secondly, based on the mechanism of the inter­action of cisplatin with DNA (Johnstone *et al.*, 2016 ▸), the reaction of complex (I) with guanine at the N7 position was proposed and investigated. Two possible reaction routes were considered:

(1) [Pt(C_9_H_6_NO)Cl(C_2_H_4_)] + guanine → [Pt(C_9_H_6_NO)(C_2_H_4_)(guanine)]^+^ + Cl^−^

(2) [Pt(C_9_H_6_NO)Cl(C_2_H_4_)] + guanine → [Pt(C_9_H_6_NO)Cl(guanine)] + C_2_H_4_

In order to know which reaction is thermodynamically more favorable, we optimized the geometric structures of the products [Pt(C_9_H_6_NO)(C_2_H_4_)(guanine)]^+^ and [Pt(C_9_H_6_NO)Cl(guanine)], as well as all species in the two reaction pathways, also followed by the frequency calculations, using the same functional and basis set as for complex (I). Then, the enthalpy changes and Gibbs free energy of the two reaction pathways were evaluated; the results are listed in Table S2.

The calculations show that reaction route (2), which corresponds to replacement of the neutral mol­ecule C_2_H_4_, which has a small negative ΔG^0^_298_ of −8.9 kJ mol^−1^, is thermodynamically more favorable than route (1), which corresponds to replacement of a Cl^−^ anion by a guanine mol­ecule with a largely positive ΔG^0^_298_ of 392.7 kJ mol^−1^.

Complex (I) could undergo a substitution reaction by replacing the Cl or C_2_H_4_ ligands with a water mol­ecule. Each of the above reaction pathways (1) and (2) can therefore take place simultaneously in two reaction steps, which are represented by the following chemical equations:

(1*a*) [Pt(C_9_H_6_NO)Cl(C_2_H_4_)] + H_2_O → [Pt(C_9_H_6_NO)(C_2_H_4_)(H_2_O)]^+^ + Cl^−^

(1*b*) [Pt(C_9_H_6_NO)(C_2_H_4_)(H_2_O)]^+^ + guanine → [Pt(C_9_H_6_NO)(C_2_H_4_)(guanine)]^+^ + H_2_O

and

(2*a*) [Pt(C_9_H_6_NO)Cl(C_2_H_4_)] + H_2_O → [Pt(C_9_H_6_NO)Cl(H_2_O)] + C_2_H_4_;

(2*b*) [Pt(C_9_H_6_NO)Cl(H_2_O)] + guanine → [Pt(C_9_H_6_NO)C(lguanine)] + H_2_O.

Using the same types of calculations as for reaction paths (1) and (2) above, the enthalpy changes and Gibbs free energies of reaction steps (1*a*), (1*b*), (2*a*) and (2*b*) were evaluated (Table S3). The results indicate that steps (1*a*) and (2*a*) with ΔG^0^_298_ = 511.6 and 36.2 kJ mol^−1^, respectively, are thermodynamically unfavorable compared to steps (1*b*) and (2*b*) with ΔG^0^_298_ = −118. 9 and −45.1 kJ mol^−1^, respectively. Substitution of the Cl ligand by water, step (1*a*), is significantly unfavorable compared to substitution of the C_2_H_4_ ligand, step (2*a*).

The transition states connecting reactants and products for reaction steps (2*a*) and (2*b*) were obtained with the same CAM B3LYP//6-31+G(d)/ccpVDZ-PP method, each of them has one imaginary frequency only, which corresponds to the stretching vibration mode where H_2_O replaces the C_2_H_4_ mol­ecule for reaction step (2*a*), and guanine replaces the H_2_O mol­ecule for reaction step (2*b*). The activation energy *E*_a_ for each reaction step was then evaluated, namely 123.7 kJ mol^−1^ for step (2*a*) (Fig. S6) and *ca* 51.4 kJ mol^−1^ for step (2*b*) (Fig. S7).

## 7. Synthesis and crystallization

A solution of 8-hy­droxy­quinoline (73 mg, 0.5 mmol) in 5 mL of ethanol was slowly added to a solution of Zeise’s salt (193 mg, 0.5 mmol) in 10 mL of water while being stirred at ambient temperature for 15 min. After continuing to stir for another 2 h, the reaction mixture was left undisturbed for 30 min. The yellow precipitate was then filtered off and washed consecutively with water (2 × 5 mL) and cold ethanol (1 × 3 mL), and finally dried under vacuum at 318 K for 3 h. The yield was 181 mg (90%). Yellow crystals suitable for X-ray diffraction were obtained by slow evaporation over 24 h from a saturated chloro­form/ethanol solution (1:1, *v*/*v*) at ambient temperature. ^1^H NMR (CDCl_3_, 500 MHz): δ 9.11 (*dd*, ^3^ *J* = 5.0 Hz, ^4^ *J* = 1.0 Hz, ^3^ *J*_PtH_ = 35 Hz, 1H, Ar-H), 8.47 (*dd*, ^3^ *J* = 8.0 Hz, ^4^ *J* = 1.0 Hz, 1H, Ar-H), 7.58 (*dd*, ^3^ *J* = 8.0 Hz, 5.0 Hz, 1H, Ar-H), 7.46 (*t*, ^3^ *J* = 8.0 Hz, 1H, Ar-H), 7.09 (*d*, ^3^ *J* = 8.0 Hz, 1H, Ar-H), 7.06 (*d*, ^3^ *J* = 8.0 Hz, 1H, Ar-H), 4.90 (*s*, ^2^ *J*_PtH_ = 60 Hz, 4H, C_2_H_4_). –ESI MS (*m*/*z*, intensity): 302, 100%, [*M* – C_9_H_6_NO – C_2_H_4_ + 2Cl]^−^. FT-IR (KBr pellet, cm^−1^): 3052, 2969 (CH), 1575, 1500 (C=C).

## 8. Refinement

Crystal data, data collection and structure refinement details are summarized in Table 3 ▸. The ethyl­ene hydrogen atoms were located in difference-Fourier maps and were refined isotropically with a C—H distance restraint of 0.93 (2) Å. Other hydrogen atoms were included as riding contributions in idealized positions with isotropic displacement parameters *U*_iso_(H) = 1.2*U*_eq_(C).

## Supplementary Material

Crystal structure: contains datablock(s) I. DOI: 10.1107/S2056989024003748/oi2006sup1.cif

Structure factors: contains datablock(s) I. DOI: 10.1107/S2056989024003748/oi2006Isup2.hkl

DFT calculation (tables, figures). DOI: 10.1107/S2056989024003748/oi2006sup3.pdf

CCDC reference: 2350722

Additional supporting information:  crystallographic information; 3D view; checkCIF report

## Figures and Tables

**Table 1:** Hydrogen-bond geometry (Å, °) *Cg*1 is the centroid of the C6–C11 ring.

*D*—H⋯*A*	*D*—H	H⋯*A*	*D*⋯*A*	*D*—H⋯*A*
C9—H9⋯O13^i^	0.93	2.58	3.462 (7)	159
C15—H15*B*⋯*Cg*1^ii^	0.93 (5)	2.95 (6)	3.645 (8)	133 (5)

Symmetry codes: (i) 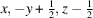 ; (ii) 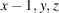 .

**Table 2:** *In vitro* cytotoxicity of complex (I) and some reference compounds, IC_50_^*a*^ in μ*M* Values highlighted in bold are the lowest values.

Complexes	KB	Lu-1	Hep-G2	MCF-7	HEK-293
[Pt(C_9_H_6_NO)Cl(C_2_H_4_)] (I)	32.1	**0.8**	**0.4**	31.1	**4.48**
Ellipticine	1.14	1.30	1.71	1.95	–
Cisplatin^*b*^	15.2	42.9	13.3	45.7	–
[Pt(C_9_H_6_NO)Cl(aryl­olefin)]^*c*^	0.39–1.45	0.44–8.17	0.38–9.58	0.61–9.04	–

Notes: (*a*) IC_50_ is the concentration of the compound required to inhibit cell growth by 50%. References: (*b*) Nguyen Thi Thanh *et al.* (2017 ▸); (*c*) Da *et al.* (2015 ▸); Thanh Chi *et al.* (2017 ▸); Nguyen Thi Thanh *et al.* (2017 ▸); Chi *et al.* (2018 ▸).

**Table 3:** Experimental details

Crystal data	
Chemical formula	[Pt(C_9_H_6_NO)Cl(C_2_H_4_)]
*M*_r_	402.74
Crystal system, space group	Monoclinic, *P*2_1_/*c*
Temperature (K)	293
*a*, *b*, *c* (Å)	7.9462 (5), 26.5977 (12), 5.1860 (2)
β (°)	100.613 (5)
*V* (Å^3^)	1077.31 (9)
*Z*	4
Radiation type	Mo *K*α
μ (mm^−1^)	13.24
Crystal size (mm)	0.35 × 0.3 × 0.05

Data collection	
Diffractometer	SuperNova, Single source at offset/far, Eos
Absorption correction	Multi-scan (*CrysAlis PRO*; Rigaku OD, 2018 ▸)
*T*_min_, *T*_max_	0.014, 0.516
No. of measured, independent and observed [*I* > 2σ(*I*)] reflections	10730, 2197, 1836
*R*_int_	0.071
(sin θ/λ)_max_ (Å^−1^)	0.625

Refinement	
*R*[*F* ^2^ > 2σ(*F* ^2^)], *wR*(*F* ^2^), *S*	0.033, 0.076, 1.09
No. of reflections	2197
No. of parameters	152
No. of restraints	4
H-atom treatment	H atoms treated by a mixture of independent and constrained refinement
Δρ_max_, Δρ_min_ (e Å^−3^)	0.93, −1.54

Computer programs: *CrysAlis PRO* (Rigaku OD, 2018 ▸), *SHELXT2014/5* (Sheldrick, 2015*a* ▸), *SHELXL* (Sheldrick, 2015*b* ▸) and *OLEX2* (Dolomanov *et al.*, 2009 ▸).

## supplementary crystallographic information

### Crystal data

**Table:**

[Pt(C_9_H_6_NO)Cl(C_2_H_4_)]	*F*(000) = 744
*M**_r_* = 402.74	*D*_x_ = 2.483 Mg m^−^^3^
Monoclinic, *P*2_1_/*c*	Mo *K*α radiation, λ = 0.71073 Å
*a* = 7.9462 (5) Å	Cell parameters from 5770 reflections
*b* = 26.5977 (12) Å	θ = 2.8–28.5°
*c* = 5.1860 (2) Å	µ = 13.24 mm^−^^1^
β = 100.613 (5)°	*T* = 293 K
*V* = 1077.31 (9) Å^3^	Plate, yellow
*Z* = 4	0.35 × 0.3 × 0.05 mm

### Data collection

**Table:**

SuperNova, Single source at offset/far, Eos diffractometer	2197 independent reflections
Radiation source: micro-focus sealed X-ray tube, SuperNova (Mo) X-ray Source	1836 reflections with *I* > 2σ(*I*)
Mirror monochromator	*R*_int_ = 0.071
Detector resolution: 15.9631 pixels mm^-1^	θ_max_ = 26.4°, θ_min_ = 2.6°
ω scans	*h* = −9→9
Absorption correction: multi-scan (CrysAlisPro; Rigaku OD, 2018)	*k* = −32→33
*T*_min_ = 0.014, *T*_max_ = 0.516	*l* = −6→6
10730 measured reflections	

### Refinement

**Table:**

Refinement on *F*^2^	4 restraints
Least-squares matrix: full	Hydrogen site location: mixed
*R*[*F*^2^ > 2σ(*F*^2^)] = 0.033	H atoms treated by a mixture of independent and constrained refinement
*wR*(*F*^2^) = 0.076	*w* = 1/[σ^2^(*F*_o_^2^) + (0.0303*P*)^2^] where *P* = (*F*_o_^2^ + 2*F*_c_^2^)/3
*S* = 1.09	(Δ/σ)_max_ = 0.001
2197 reflections	Δρ_max_ = 0.93 e Å^−^^3^
152 parameters	Δρ_min_ = −1.54 e Å^−^^3^

### Special details

**Table:**

Geometry. All esds (except the esd in the dihedral angle between two l.s. planes) are estimated using the full covariance matrix. The cell esds are taken into account individually in the estimation of esds in distances, angles and torsion angles; correlations between esds in cell parameters are only used when they are defined by crystal symmetry. An approximate (isotropic) treatment of cell esds is used for estimating esds involving l.s. planes.

### Fractional atomic coordinates and isotropic or equivalent isotropic displacement parameters (Å^2^)

**Table:**

	*x*	*y*	*z*	*U*_iso_*/*U*_eq_	
Pt1	0.38307 (3)	0.36276 (2)	0.72718 (5)	0.03174 (12)	
N2	0.5701 (7)	0.40559 (19)	0.6148 (10)	0.0357 (13)	
C3	0.6158 (9)	0.4525 (3)	0.6764 (14)	0.0418 (17)	
H3	0.563022	0.469543	0.796241	0.050*	
C4	0.7414 (11)	0.4772 (3)	0.5669 (16)	0.054 (2)	
H4	0.768667	0.510536	0.609190	0.065*	
C5	0.8231 (10)	0.4519 (3)	0.3978 (14)	0.0471 (19)	
H5	0.905763	0.468137	0.322614	0.057*	
C6	0.7829 (9)	0.4010 (2)	0.3358 (12)	0.0354 (15)	
C7	0.8637 (10)	0.3712 (3)	0.1672 (14)	0.0432 (19)	
H7	0.950317	0.384603	0.089508	0.052*	
C8	0.8121 (9)	0.3227 (3)	0.1209 (13)	0.0411 (17)	
H8	0.863222	0.303451	0.006866	0.049*	
C9	0.6837 (9)	0.3004 (2)	0.2395 (11)	0.0348 (15)	
H9	0.654280	0.266856	0.206976	0.042*	
C10	0.6030 (8)	0.3283 (2)	0.4021 (11)	0.0283 (14)	
C11	0.6566 (9)	0.3790 (2)	0.4520 (11)	0.0310 (15)	
Cl12	0.2795 (3)	0.42475 (7)	0.9633 (4)	0.0571 (6)	
O13	0.4778 (6)	0.31029 (15)	0.5162 (8)	0.0339 (11)	
C14	0.2577 (10)	0.3057 (3)	0.9145 (14)	0.0394 (18)	
C15	0.1476 (10)	0.3214 (3)	0.6918 (15)	0.0408 (17)	
H15A	0.140 (10)	0.302 (2)	0.540 (9)	0.07 (2)*	
H14A	0.227 (8)	0.319 (2)	1.066 (8)	0.031 (18)*	
H15B	0.055 (6)	0.343 (2)	0.675 (12)	0.037 (19)*	
H14B	0.338 (9)	0.280 (2)	0.914 (16)	0.10 (3)*	

### Atomic displacement parameters (Å^2^)

**Table:**

	*U*^11^	*U*^22^	*U*^33^	*U*^12^	*U*^13^	*U*^23^
Pt1	0.0326 (2)	0.03370 (18)	0.03123 (17)	0.00135 (10)	0.01189 (13)	0.00116 (9)
N2	0.046 (4)	0.032 (3)	0.033 (3)	0.000 (3)	0.017 (3)	0.000 (2)
C3	0.038 (5)	0.040 (4)	0.047 (4)	−0.002 (3)	0.007 (3)	−0.004 (3)
C4	0.057 (6)	0.036 (4)	0.070 (5)	−0.009 (4)	0.014 (5)	−0.015 (4)
C5	0.034 (5)	0.052 (5)	0.058 (5)	−0.016 (4)	0.017 (4)	0.001 (4)
C6	0.032 (4)	0.039 (4)	0.036 (3)	−0.002 (3)	0.007 (3)	0.004 (3)
C7	0.040 (5)	0.052 (5)	0.039 (4)	−0.004 (3)	0.012 (3)	−0.001 (3)
C8	0.033 (4)	0.053 (5)	0.039 (4)	−0.003 (3)	0.011 (3)	−0.008 (3)
C9	0.039 (4)	0.032 (3)	0.034 (3)	−0.001 (3)	0.008 (3)	−0.003 (3)
C10	0.021 (4)	0.036 (3)	0.029 (3)	−0.001 (3)	0.007 (3)	0.003 (3)
C11	0.032 (4)	0.032 (3)	0.028 (3)	−0.005 (3)	0.002 (3)	−0.001 (3)
Cl12	0.0660 (15)	0.0478 (11)	0.0678 (13)	0.0010 (10)	0.0389 (12)	−0.0107 (9)
O13	0.036 (3)	0.028 (2)	0.043 (2)	−0.006 (2)	0.021 (2)	−0.0002 (19)
C14	0.041 (5)	0.044 (4)	0.038 (4)	0.005 (4)	0.021 (4)	0.006 (3)
C15	0.025 (4)	0.051 (5)	0.045 (4)	0.003 (3)	0.003 (3)	0.001 (4)

### Geometric parameters (Å, º)

**Table:**

Pt1—N2	2.041 (5)	C6—C11	1.390 (9)
Pt1—Cl12	2.2951 (18)	C7—H7	0.9300
Pt1—O13	2.004 (4)	C7—C8	1.363 (9)
Pt1—C14	2.143 (7)	C8—H8	0.9300
Pt1—C15	2.149 (8)	C8—C9	1.414 (9)
N2—C3	1.321 (8)	C9—H9	0.9300
N2—C11	1.378 (8)	C9—C10	1.368 (8)
C3—H3	0.9300	C10—C11	1.424 (8)
C3—C4	1.399 (10)	C10—O13	1.336 (7)
C4—H4	0.9300	C14—C15	1.379 (10)
C4—C5	1.362 (10)	C14—H14A	0.93 (2)
C5—H5	0.9300	C14—H14B	0.94 (2)
C5—C6	1.414 (9)	C15—H15A	0.93 (2)
C6—C7	1.418 (9)	C15—H15B	0.93 (2)
			
N2—Pt1—Cl12	95.90 (15)	C8—C7—H7	120.6
N2—Pt1—C14	161.5 (3)	C7—C8—H8	118.7
N2—Pt1—C15	158.4 (3)	C7—C8—C9	122.6 (6)
O13—Pt1—N2	82.32 (19)	C9—C8—H8	118.7
O13—Pt1—Cl12	178.18 (12)	C8—C9—H9	120.1
O13—Pt1—C14	90.4 (2)	C10—C9—C8	119.9 (6)
O13—Pt1—C15	90.3 (2)	C10—C9—H9	120.1
C14—Pt1—Cl12	91.4 (2)	C9—C10—C11	117.8 (6)
C14—Pt1—C15	37.5 (3)	O13—C10—C9	123.4 (6)
C15—Pt1—Cl12	91.2 (2)	O13—C10—C11	118.8 (5)
C3—N2—Pt1	129.8 (5)	N2—C11—C6	122.1 (6)
C3—N2—C11	119.1 (6)	N2—C11—C10	115.5 (5)
C11—N2—Pt1	111.1 (4)	C6—C11—C10	122.3 (6)
N2—C3—H3	119.0	C10—O13—Pt1	112.2 (4)
N2—C3—C4	122.0 (7)	Pt1—C14—H14A	109 (4)
C4—C3—H3	119.0	Pt1—C14—H14B	98 (5)
C3—C4—H4	120.4	C15—C14—Pt1	71.5 (4)
C5—C4—C3	119.3 (7)	C15—C14—H14A	112 (4)
C5—C4—H4	120.4	C15—C14—H14B	123 (5)
C4—C5—H5	119.8	H14A—C14—H14B	124 (7)
C4—C5—C6	120.3 (7)	Pt1—C15—H15A	106 (5)
C6—C5—H5	119.8	Pt1—C15—H15B	110 (4)
C5—C6—C7	124.4 (6)	C14—C15—Pt1	71.0 (4)
C11—C6—C5	117.0 (6)	C14—C15—H15A	118 (5)
C11—C6—C7	118.5 (6)	C14—C15—H15B	130 (4)
C6—C7—H7	120.6	H15A—C15—H15B	110 (6)
C8—C7—C6	118.8 (6)		
			
Pt1—N2—C3—C4	−175.9 (6)	C7—C6—C11—N2	−178.9 (6)
Pt1—N2—C11—C6	175.9 (5)	C7—C6—C11—C10	−1.7 (10)
Pt1—N2—C11—C10	−1.5 (7)	C7—C8—C9—C10	1.9 (11)
N2—C3—C4—C5	−2.1 (12)	C8—C9—C10—C11	−2.0 (9)
C3—N2—C11—C6	−4.5 (10)	C8—C9—C10—O13	178.3 (6)
C3—N2—C11—C10	178.1 (6)	C9—C10—C11—N2	179.4 (6)
C3—C4—C5—C6	−0.7 (12)	C9—C10—C11—C6	2.0 (9)
C4—C5—C6—C7	−178.5 (7)	C9—C10—O13—Pt1	−177.4 (5)
C4—C5—C6—C11	0.8 (11)	C11—N2—C3—C4	4.6 (11)
C5—C6—C7—C8	−179.3 (7)	C11—C6—C7—C8	1.4 (11)
C5—C6—C11—N2	1.8 (10)	C11—C10—O13—Pt1	2.9 (7)
C5—C6—C11—C10	179.0 (6)	O13—C10—C11—N2	−0.9 (8)
C6—C7—C8—C9	−1.5 (11)	O13—C10—C11—C6	−178.3 (6)

### Hydrogen-bond geometry (Å, º)

*Cg*1 is the centroid of the C6–C11 ring.

**Table:**

*D*—H···*A*	*D*—H	H···*A*	*D*···*A*	*D*—H···*A*
C9—H9···O13^i^	0.93	2.58	3.462 (7)	159
C15—H15*B*···*Cg*1^ii^	0.93 (5)	2.95 (6)	3.645 (8)	133 (5)

Symmetry codes: (i) *x*, −*y*+1/2, *z*−1/2; (ii) *x*−1, *y*, *z*.
