# Supplementary material for: Synthesis, crystal structure and anti­cancer activity of the complex chlorido­(η2-ethyl­ene)(quinolin-8-olato-κ2 N,O)platinum(II) by experimental and theoretical methods
Source: Acta Crystallogr E Crystallogr Commun. 2024 Apr 30;80(Pt 5):550–4. doi: 10.1107/S2056989024003748 (PMC11074582; doi:10.1107/S2056989024003748)
Supplement: Supplementary file 3 [file e-80-00550-sup3.pdf]

Synthesis, crystal structure and anticancer activity of the complex [PtCl(C_9H_6NO)(C_2H_4)] by experimental and theoretical methods

Nguyen Thi Thanh Chi,[a] Ngo Tuan Cuong,[a] Tran Thu Trang,[a] Pham Van Thong,[ab] Nguyen Thi Bang Linh,[a] Nguyen Thi Khanh Ly[a] and Luc Van Meervelt[c]*

[a]Department of Chemistry, Hanoi National University of Education, 136 Xuan Thuy, Cau Giay, Hanoi, Vietnam, [b]R&D Center, Vietnam Education and Technology Transfer JSC, Hanoi, Vietnam, and [c]Department of Chemistry, KU Leuven, Biomolecular Architecture, Celestijnenlaan 200F, Leuven (Heverlee), B-3001, Belgium

Supporting Information

I. Experimental ESI-MS, IR, ^{1}H-NMR and NOESY NMR spectrum of complex [PtCl(C_9H_6NO)(C_2H_4)].

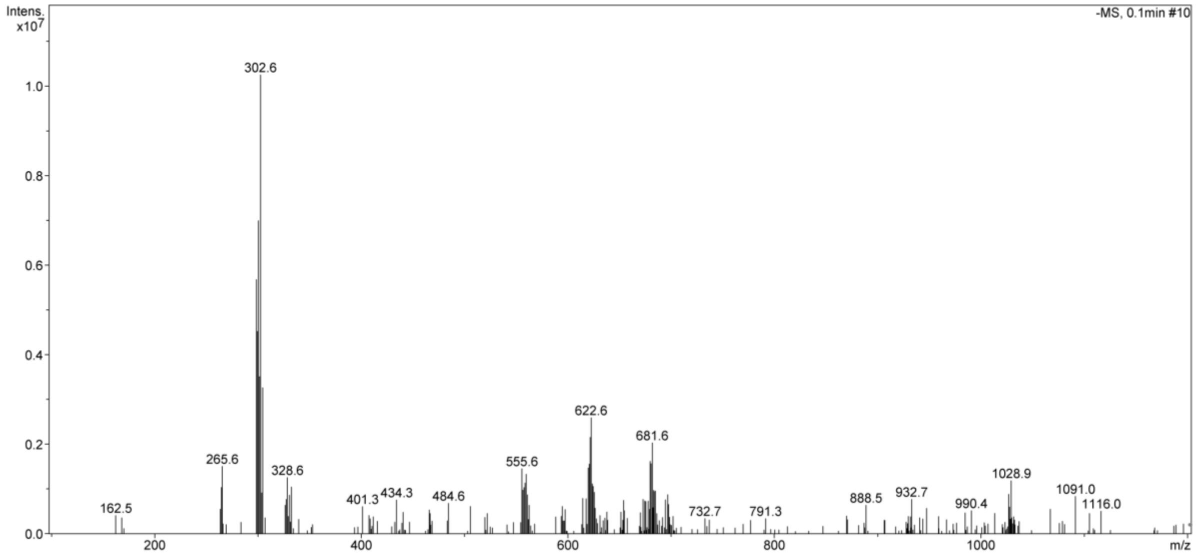

Fig. S1. Negative-mode ESI-MS spectrum of complex [PtCl(C_9H_6NO)(C_2H_4)].

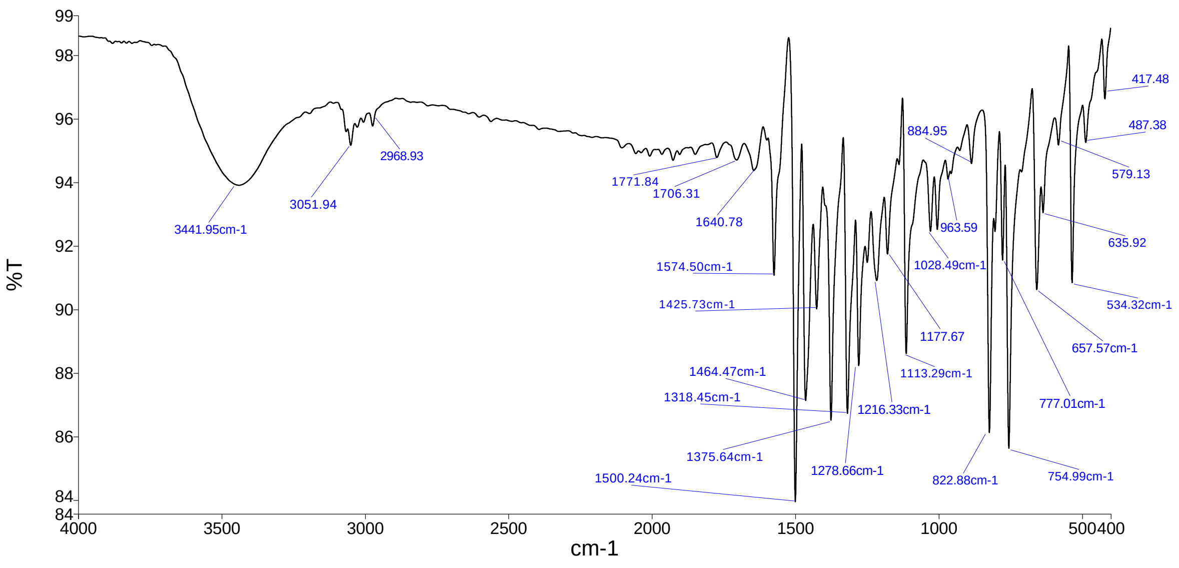

Fig. S2. IR spectrum of complex [PtCl(C_9H_6NO)(C_2H_4)].

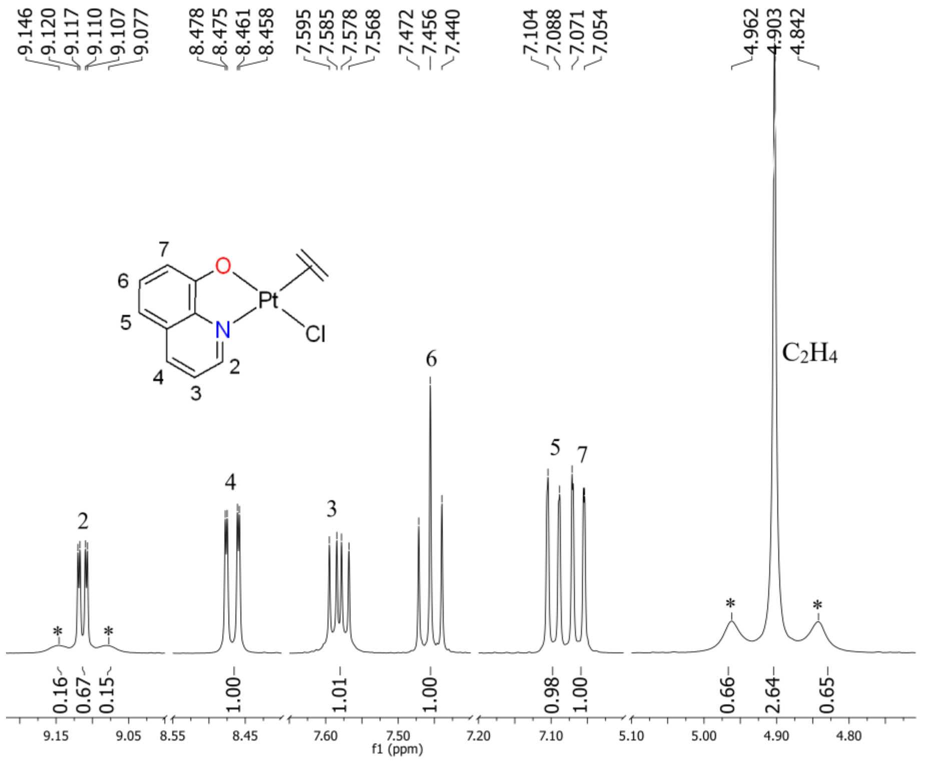

Fig. S3. ^{1}H NMR spectrum of complex [PtCl(C_9H_6NO)(C_2H_4)].

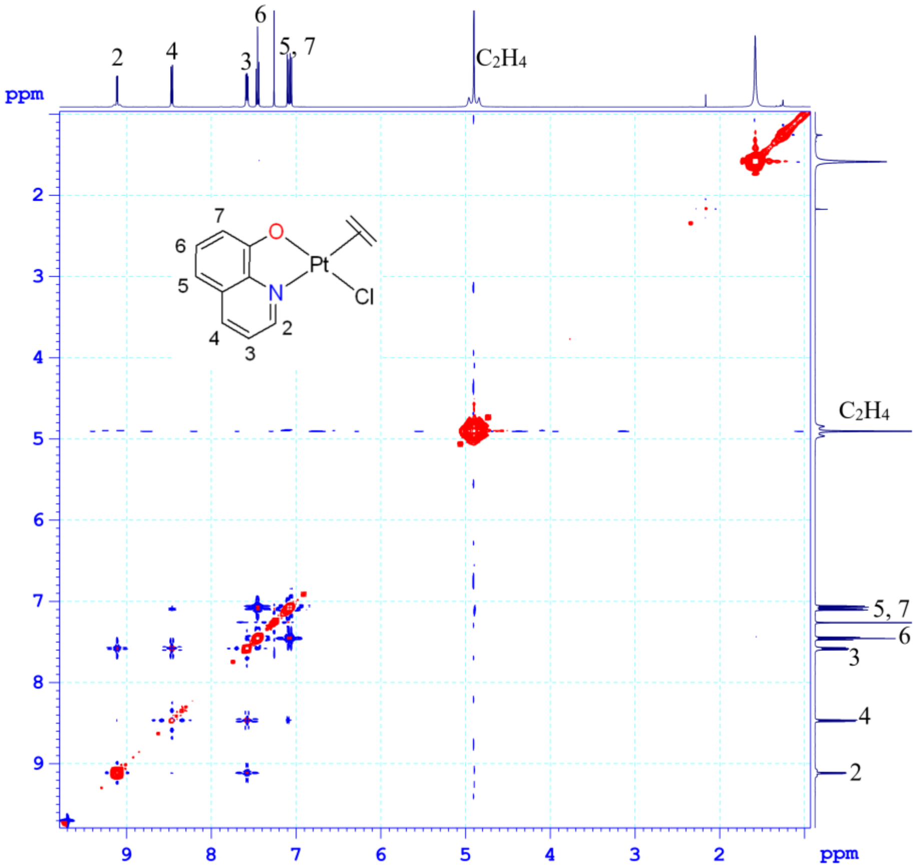

Fig. S4. NOESY NMR spectrum of complex [PtCl(C_9H_6NO)(C_2H_4)].

II. DFT calculation results.

II.1. The structure of complex (I)

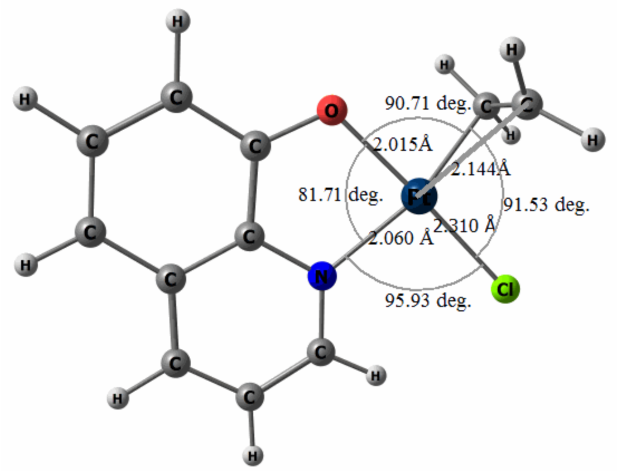

Fig. S5. The structure of complex (I) which is optimized using the CAM-B3LYP/6-311+G(d)/Aug-cc-pVDZ-PP combination of functional and basis set.

Table S1. Selected bond lengths (Å) and angles (°) for complex (I) determined by DFT and XRD (*Cg* is the centroid of C=C olefin).

Complexes	Pt—N	Pt—Cl	Pt—O	Pt—*Cg*	C=C$_{olefinic}$	N—Pt—O	N—Pt—Cl	O—Pt—Cl
XRD	2.041(5)	2.2951(18)	2.004(4)	2.033(6)	1.379(10)	82.32(19)	95.90(15)	178.18(72)
DFT	2.060	2.310	2.015	2.029	1.386	81.71	95.93	177.64

II.2. The DFT calculation results on energetics of the investigated reactions

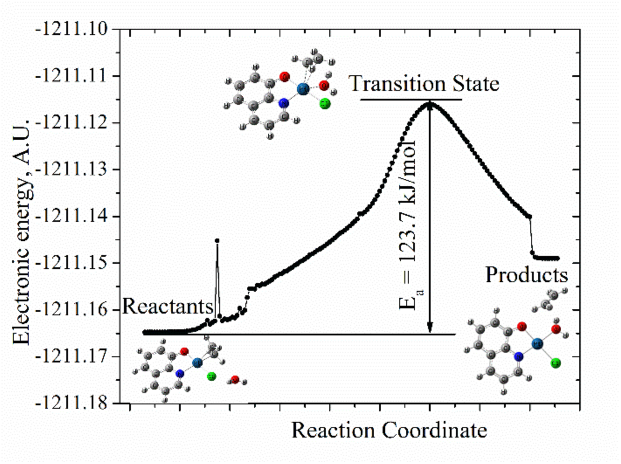

Figure S6. The reactants, transition state, products and activation energy E_a for the reaction step (2a).

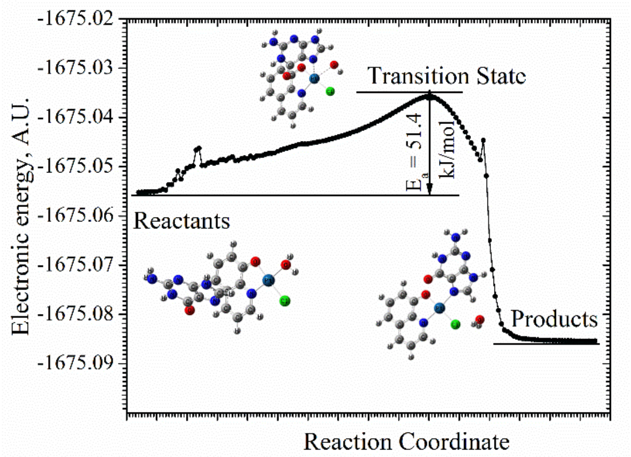

Figure S7. The reactants, transition state, products and activation energy E_a for the reaction step (2b).

Table S2. Calculated enthalpy change and free Gibbs energy for reaction pathways (1) and (2).

(1) $[PtCl(C_9H_6NO)(C_2H_4)]$ + guanine → $[Pt(C_9H_6NO)(C_2H_4)(guanine)]^+ + Cl^-$
(2) $[PtCl(C_9H_6NO)(C_2H_4)]$ + guanine → $[PtCl(C_9H_6NO)(guanine)] + C_2H_4$

	Reaction (1)	Reaction (2)
ΔH^0_{298}, kJ/mol	377.6	-10.8
ΔG^0_{298}, kJ/mol	392.7	-8.9

Table S3. Calculated enthalpy change and free Gibbs energy for reaction steps (1a), (1b), (2a) and (2b).

(1a) $[PtCl(C_9H_6NO)(C_2H_4)] + H_2O \rightarrow [Pt(C_9H_6NO)(C_2H_4)(H_2O)]^+ + Cl^-$;
(1b) $[Pt(C_9H_6NO)(C_2H_4)(H_2O)]^+$ + Guanine → $[Pt(C_9H_6NO)(C_2H_4)(Guanine)]^+ + H_2O$
and
(2a) $[PtCl(C_9H_6NO)(C_2H_4)] + H_2O \rightarrow [PtCl(C_9H_6NO)(H_2O)] + C_2H_4$;
(2b) $[PtCl(C_9H_6NO)(H_2O)]$ + Guanine → $[PtCl(C_9H_6NO)Guanine] + H_2O$

Reaction steps	Step (1a)	Step (1b)	Step (2a)	Step (2b)
ΔH^0_{298}, kJ/mol	503.0	-125.4	40.7	-51.5
ΔG^0_{298}, kJ/mol	511.6	-118.9	36.2	-45.1

II.3. Cartesian Coordinates of the complex compound in the reaction steps (2a,b)

Reactants $[PtCl(C_9H_6NO)(C_2H_4)] + H_2O$ of step (2a)

Pt	0.800988000000	-0.239146000000	-0.192974000000
Cl	2.247858000000	1.561338000000	-0.449779000000
C	2.350837000000	-1.612843000000	0.426811000000
H	1.826630000000	-2.376074000000	0.991082000000
H	3.122632000000	-1.061386000000	0.953472000000
C	2.274011000000	-1.608252000000	-0.955258000000
H	2.986330000000	-1.042853000000	-1.544110000000
H	1.714631000000	-2.374803000000	-1.479703000000
C	-2.010569000000	0.108386000000	0.085658000000
C	-1.038618000000	2.217294000000	-0.096674000000
C	-1.769540000000	-1.293280000000	0.114676000000
C	-3.308897000000	0.646476000000	0.200280000000
C	-2.297754000000	2.829701000000	0.010866000000
H	-0.126733000000	2.788673000000	-0.214333000000
C	-2.862603000000	-2.126883000000	0.261372000000
C	-4.396684000000	-0.240129000000	0.348288000000
C	-3.416373000000	2.054919000000	0.157342000000

H	-2.360876000000	3.909736000000	-0.024021000000
C	-4.156808000000	-1.592130000000	0.375798000000
H	-2.702078000000	-3.197961000000	0.286956000000
H	-5.402345000000	0.153157000000	0.438414000000
H	-4.395864000000	2.513990000000	0.242106000000
H	-4.989720000000	-2.277394000000	0.489972000000
N	-0.907009000000	0.908058000000	-0.059893000000
O	-0.529746000000	-1.729657000000	0.001697000000
O	4.362676000000	0.536801000000	1.797744000000
H	5.272363000000	0.845718000000	1.789102000000
H	3.847982000000	1.116686000000	1.218079000000

Transition state of the step (2a)

Pt	-0.918900000000	-0.067516000000	-0.131295000000
Cl	-2.324151000000	1.792075000000	-0.092424000000
C	-2.646385000000	-1.552779000000	1.354306000000
H	-2.443450000000	-2.551254000000	0.982008000000
H	-3.608160000000	-1.109739000000	1.121443000000
C	-1.775923000000	-0.924608000000	2.154685000000
H	-2.008205000000	0.036455000000	2.597015000000
H	-0.851736000000	-1.398128000000	2.463362000000
C	1.894157000000	0.024455000000	-0.024456000000
C	1.051449000000	2.189342000000	0.139522000000
C	1.555803000000	-1.349677000000	-0.154015000000
C	3.229080000000	0.466743000000	0.060694000000
C	2.356544000000	2.705626000000	0.233250000000
H	0.176729000000	2.825287000000	0.160291000000
C	2.594210000000	-2.263678000000	-0.199473000000
C	4.257316000000	-0.497787000000	0.010213000000
C	3.430233000000	1.861096000000	0.193534000000
H	2.485429000000	3.775928000000	0.333420000000
C	3.925603000000	-1.825758000000	-0.117921000000
H	2.361469000000	-3.316863000000	-0.301082000000
H	5.292297000000	-0.182727000000	0.072404000000
H	4.441107000000	2.248638000000	0.262882000000
H	4.716031000000	-2.567649000000	-0.158136000000
N	0.834637000000	0.897037000000	0.015162000000
O	0.281995000000	-1.675645000000	-0.219000000000
O	-2.559592000000	-0.881521000000	-1.612673000000
H	-3.082258000000	-1.662063000000	-1.406059000000
H	-3.148702000000	-0.116727000000	-1.670149000000

Products $[PtCl(C_9H_6NO)(H_2O)]^0$ + C_2H_4 of step (2a)

Pt	0.810738000000	-0.433660000000	-0.162481000000
Cl	1.665442000000	-2.568180000000	0.120004000000
C	3.705596000000	3.083363000000	0.383478000000
H	3.470612000000	3.311825000000	-0.651411000000
H	4.747724000000	3.167460000000	0.674880000000
C	2.757817000000	2.747205000000	1.251279000000
H	2.992389000000	2.537433000000	2.290217000000
H	1.716627000000	2.668094000000	0.953823000000
C	-1.888030000000	0.302227000000	-0.000458000000
C	-1.672797000000	-1.992887000000	0.339952000000
C	-1.186696000000	1.510775000000	-0.261648000000
C	-3.288289000000	0.258684000000	0.144584000000
C	-3.064177000000	-2.115045000000	0.495504000000
H	-1.006577000000	-2.841856000000	0.408658000000
C	-1.933093000000	2.670545000000	-0.371181000000
C	-4.010869000000	1.464973000000	0.026573000000
C	-3.861604000000	-1.008549000000	0.400057000000
H	-3.481177000000	-3.094897000000	0.689821000000
C	-3.329706000000	2.631491000000	-0.225723000000
H	-1.424073000000	3.605288000000	-0.574000000000
H	-5.089115000000	1.457787000000	0.133873000000
H	-4.936811000000	-1.091585000000	0.518337000000
H	-3.883994000000	3.559325000000	-0.318253000000
N	-1.111312000000	-0.825468000000	0.101215000000
O	0.123170000000	1.455658000000	-0.380454000000
O	2.792321000000	0.098680000000	-0.496113000000
H	3.083682000000	0.893963000000	-0.015324000000
H	3.346960000000	-0.661398000000	-0.267648000000

Reactants $[PtCl(C_9H_6NO)(H_2O)]$ + Guanine of step (2b)

C	-1.507136000000	0.528875000000	-0.402798000000
C	-1.814430000000	-0.677081000000	1.567512000000
C	-1.430037000000	0.443888000000	-1.819070000000
C	-1.386572000000	1.746881000000	0.287779000000
C	-1.720540000000	0.502021000000	2.320983000000
H	-1.976038000000	-1.644073000000	2.023234000000
C	-1.223091000000	1.616453000000	-2.520237000000
C	-1.170425000000	2.922485000000	-0.464965000000
C	-1.503823000000	1.698327000000	1.696224000000
H	-1.799428000000	0.438812000000	3.398291000000
C	-1.094656000000	2.837642000000	-1.833530000000
H	-1.162738000000	1.578898000000	-3.601211000000
H	-1.063315000000	3.869780000000	0.049438000000

H	-1.389952000000	2.609313000000	2.268781000000
H	-0.933208000000	3.740294000000	-2.413808000000
N	-1.711137000000	-0.659030000000	0.254468000000
O	-1.560367000000	-0.745156000000	-2.376581000000
C	2.350190000000	1.498385000000	1.097614000000
C	2.324576000000	-0.553235000000	0.556897000000
C	3.478302000000	1.302672000000	0.321097000000
C	2.071872000000	2.818851000000	1.582922000000
H	2.064241000000	-1.594000000000	0.434391000000
H	4.130903000000	-0.487706000000	-0.597245000000
C	4.179459000000	3.398209000000	0.393879000000
H	3.006125000000	4.644214000000	1.548051000000
N	1.643205000000	0.322930000000	1.227974000000
N	3.455841000000	-0.016783000000	-0.016468000000
N	4.413094000000	2.205615000000	-0.061899000000
N	3.094190000000	3.713725000000	1.163212000000
N	5.072688000000	4.412651000000	0.151935000000
H	5.777426000000	4.167853000000	-0.526960000000
H	4.706356000000	5.345406000000	0.039318000000
O	1.156230000000	3.242209000000	2.256176000000
Pt	-1.859565000000	-2.178509000000	-0.999419000000
Cl	-2.234935000000	-3.910851000000	0.502642000000
H	-2.353143000000	-4.475598000000	-1.954973000000
H	-2.608758000000	-3.461428000000	-3.147911000000
O	-2.011604000000	-3.700868000000	-2.430050000000

Transition state of step (2b)

C	-2.305544000000	1.156592000000	-0.228255000000
C	-2.684930000000	0.001930000000	1.757002000000
C	-1.566745000000	1.168517000000	-1.442243000000
C	-3.216084000000	2.175131000000	0.110873000000
C	-3.603284000000	0.984683000000	2.167065000000
H	-2.451825000000	-0.859856000000	2.366519000000
C	-1.782163000000	2.224675000000	-2.312434000000
C	-3.404489000000	3.234337000000	-0.802920000000
C	-3.870656000000	2.053647000000	1.359150000000
H	-4.087719000000	0.871008000000	3.128767000000
C	-2.695564000000	3.237782000000	-1.981006000000
H	-1.233054000000	2.255157000000	-3.246065000000
H	-4.101172000000	4.029987000000	-0.565728000000
H	-4.579329000000	2.815851000000	1.665678000000
H	-2.841375000000	4.051579000000	-2.683943000000

N	-2.065264000000	0.086667000000	0.598276000000
O	-0.727860000000	0.184114000000	-1.665252000000
C	2.519181000000	0.108283000000	0.087502000000
C	2.689558000000	-1.722750000000	-0.989178000000
C	3.839715000000	0.025897000000	-0.306372000000
C	2.090753000000	1.255906000000	0.836686000000
H	2.468522000000	-2.658736000000	-1.475366000000
H	4.774837000000	-1.511889000000	-1.419473000000
C	4.472993000000	1.946166000000	0.589329000000
H	2.977464000000	2.931758000000	1.618821000000
N	1.823231000000	-0.996929000000	-0.349817000000
N	3.937521000000	-1.149866000000	-0.992272000000
N	4.846405000000	0.906563000000	-0.096569000000
N	3.196444000000	2.134728000000	1.036980000000
N	5.392837000000	2.899569000000	0.933749000000
H	6.281670000000	2.800255000000	0.469305000000
H	5.083136000000	3.850345000000	1.054793000000
O	1.010917000000	1.551545000000	1.289925000000
Pt	-0.703415000000	-1.138089000000	-0.146785000000
Cl	-0.735988000000	-2.755886000000	1.539283000000
H	-0.129991000000	-3.644319000000	-0.638614000000
H	-0.571976000000	-3.158224000000	-2.054597000000
O	0.088728000000	-3.038388000000	-1.364963000000

Products $[PtCl(C_9H_6NO)Guanine]^0 + H_2O$ of step (2b)

C	2.791319000000	0.905233000000	0.155951000000
C	3.246685000000	-1.061780000000	-0.999854000000
C	1.781210000000	1.672269000000	0.805129000000
C	4.126439000000	1.347819000000	0.061203000000
C	4.595615000000	-0.690094000000	-1.136990000000
H	2.860573000000	-1.989649000000	-1.400346000000
C	2.159763000000	2.886802000000	1.354576000000
C	4.466516000000	2.591112000000	0.634831000000
C	5.030776000000	0.495681000000	-0.614046000000
H	5.269894000000	-1.355982000000	-1.660630000000
C	3.489058000000	3.326716000000	1.262316000000
H	1.409614000000	3.489519000000	1.852405000000
H	5.487010000000	2.950633000000	0.572975000000
H	6.069050000000	0.795005000000	-0.713232000000
H	3.747554000000	4.283899000000	1.703143000000
N	2.381933000000	-0.291550000000	-0.374708000000
O	0.561697000000	1.194345000000	0.853058000000
C	-2.536057000000	0.253516000000	-0.033347000000
C	-2.161466000000	-1.537491000000	1.120154000000

C	-3.734436000000	-0.097666000000	0.545674000000
C	-2.492994000000	1.379044000000	-0.926717000000
H	-1.685788000000	-2.402106000000	1.568340000000
H	-4.152644000000	-1.752249000000	1.803116000000
C	-4.907193000000	1.560784000000	-0.330371000000
H	-3.838735000000	2.750154000000	-1.638819000000
N	-1.569509000000	-0.662654000000	0.344941000000
N	-3.477673000000	-1.228605000000	1.268915000000
N	-4.932894000000	0.518561000000	0.449171000000
N	-3.783176000000	1.986554000000	-0.978556000000
N	-6.056532000000	2.257997000000	-0.565529000000
H	-6.831481000000	2.003799000000	0.025968000000
H	-6.001942000000	3.233001000000	-0.810475000000
O	-1.597805000000	1.824690000000	-1.597088000000
Pt	0.412261000000	-0.590413000000	-0.070592000000
Cl	0.308062000000	-2.684497000000	-1.098683000000
H	-0.175294000000	-3.879233000000	0.799179000000
H	0.162602000000	-4.289887000000	2.263164000000
O	-0.561427000000	-4.069289000000	1.671323000000
